# Infection of *Helicobacter pylori* contributes to the progression of gastric cancer through ferroptosis

**DOI:** 10.1038/s41420-024-02253-3

**Published:** 2024-12-02

**Authors:** Yun Liu, Renjie Miao, Jinxuan Xia, Yong Zhou, Jun Yao, Shihe Shao

**Affiliations:** 1 Department of Gastroenterology, Institute of Digestive Disease, The Affiliated People’s Hospital, Jiangsu University, Zhenjiang, Jiangsu China; 2 School of Medicine, Jiangsu University, Zhenjiang, Jiangsu China; 3 Department of Clinical laboratory, Affiliated Third Hospital of Zhenjiang to Jiangsu University, Zhenjiang, Jiangsu China; 4 Zhenjiang Mental Health Center, Jiangsu, China

**Keywords:** Cell death, Gastric cancer

## Abstract

*Helicobacter pylori* (*H. pylori*) is a gram-negative pathogen that colonizes gastric epithelial cells, and its chronic infection is the primary risk factor for the development of gastric cancer (GC). Ferroptosis is an iron-dependent form of cell death characterized by intracellular lipid peroxide accumulation and reactive oxygen species (ROS) imbalance. There is evidence suggesting that pathogens can manipulate ferroptosis to facilitate their replication, transmission, and pathogenesis. However, the interaction between ferroptosis and *H. pylori* infection requires further elucidation. We reviewed the mechanism of ferroptosis and found that *H. pylori* virulence factors such as cytotoxin-associated gene A (CagA), vacuolating cytotoxin A (VacA), neutrophil-activating protein A (NapA), superoxide dismutase B (SodB), γ-glutamyl transpeptidase (gGT), lipopolysaccharide (LPS), and outer inflammatory protein A (OipA) affected glutathione (GSH), ROS, and lipid oxidation to regulate ferroptosis. It also affected the progression of GC by regulating ferroptosis-related indicators through abnormal gene expression after *H. pylori* infected gastric mucosa cells. Finally, we discuss the potential application value of ferroptosis inducers, inhibitors and other drugs in treating *H. pylori*-infected GC patients while acknowledging that their interactions are still not fully understood.

## FACTS

- *H. pylori* virulence factors affected glutathione, ROS and lipid oxidation to regulate ferroptosis.
- *H. pylori* infected gastric mucosa cells regulating ferroptosis-related indicators affected the progression of GC.
- Targeting ferroptosis is the potential application value in treating *H. pylori*-infected GC patients.

## OPEN QUESTIONS

- Is there a role for ferroptosis in acute and chronic *H. pylori* infection of gastric mucosa?
- Which factors affect the process of ferroptosis in gastric mucosa cells infected with *H. pylori*?
- What is the mechanism by which *H. pylori* infected gastric cancer cells regulate ferroptosis and affect the disease?
- What is the therapeutic potential of ferroptosis-related targets to modulate *H. pylori* infection in gastric cancer?

## Introduction

*Helicobacter pylori* (*H. pylori*) is a gram-negative bacterium that was first isolated from the stomach epithelia of patients with active chronic gastritis in 1982 by Barry Marshall and Robin Warren [1]. *H. pylori* has been classified as a Group I carcinogenic pathogen by the International Agency for Research on Cancer and the World Health Organization, and its infection is considered an important risk factor for gastric cancer (GC) [2–4]. The global prevalence of *H. pylori* infection has declined during the last 3 decades in adults, but not in children and adolescents [5]. *H. pylori* could colonize gastric epithelial cells due to its main components enabling it to survive in an acidic stomach environment and attach to host cells through the interaction of microbial adhesins with host cell receptors through flagellate-mediated motility; its virulence factors such as cytotoxin-associated gene A (CagA) and vacuolating cytotoxin A (VacA), are implicated in the development of GC [6, 7]. In addition, *H. pylori* infection can mediate GC cells inflammation, epithelial-mesenchymal transition, migration, invasion, IL-8 secretion, and hummingbird-like changes in GC cells leading to disease progression [8–11]. *H. pylori* infected gastric epithelium could cause the accumulation of neutrophils, which induces the production of ROS [12–14]. The release of ROS can lead to a decrease in cellular glutathione (GSH) concentrations in *H. pylori*-infected gastric epithelial cells [15]. Furthermore, GC-derived *H. pylori*-induced oxidative stress is more potent and has suppressive effects on the host’s GSH-related defense systems [16]. These studies demonstrate that *H. pylori* infection can result in ROS release and GSH depletion, which are key characteristics of the occurrence of ferroptosis.

Ferroptosis is a type of non-apoptotic cell death triggered by the selective depletion of small molecules by erastin, leading to the demise of specific tumor cells or activation under certain conditions [17]. Depletion of GSH, excess iron ions, dysregulated lipid metabolism, and imbalanced ROS are key characteristics of ferroptosis [18, 19]. The presence of *H. pylori* influences ROS modulation and may make GC cells inherently susceptible to ferroptosis. Numerous studies have highlighted the critical role of ferroptosis in GC development, progression, treatment, and prognosis [20–22]. This review summarizes the mechanisms of CagA, VacA, NapA, SodB, gGT, LPS and OipA or *H. pylori* regulate ferroptosis. It also discusses how *H. pylori* regulates ferroptosis to promote infection-induced carcinogenesis. Additionally, it provides an overview of clinical trials involving ferroptosis-targeting drugs used in *H. pylori*-infected GC patients.

## Overview of ferroptosis

**Fig. 1:**
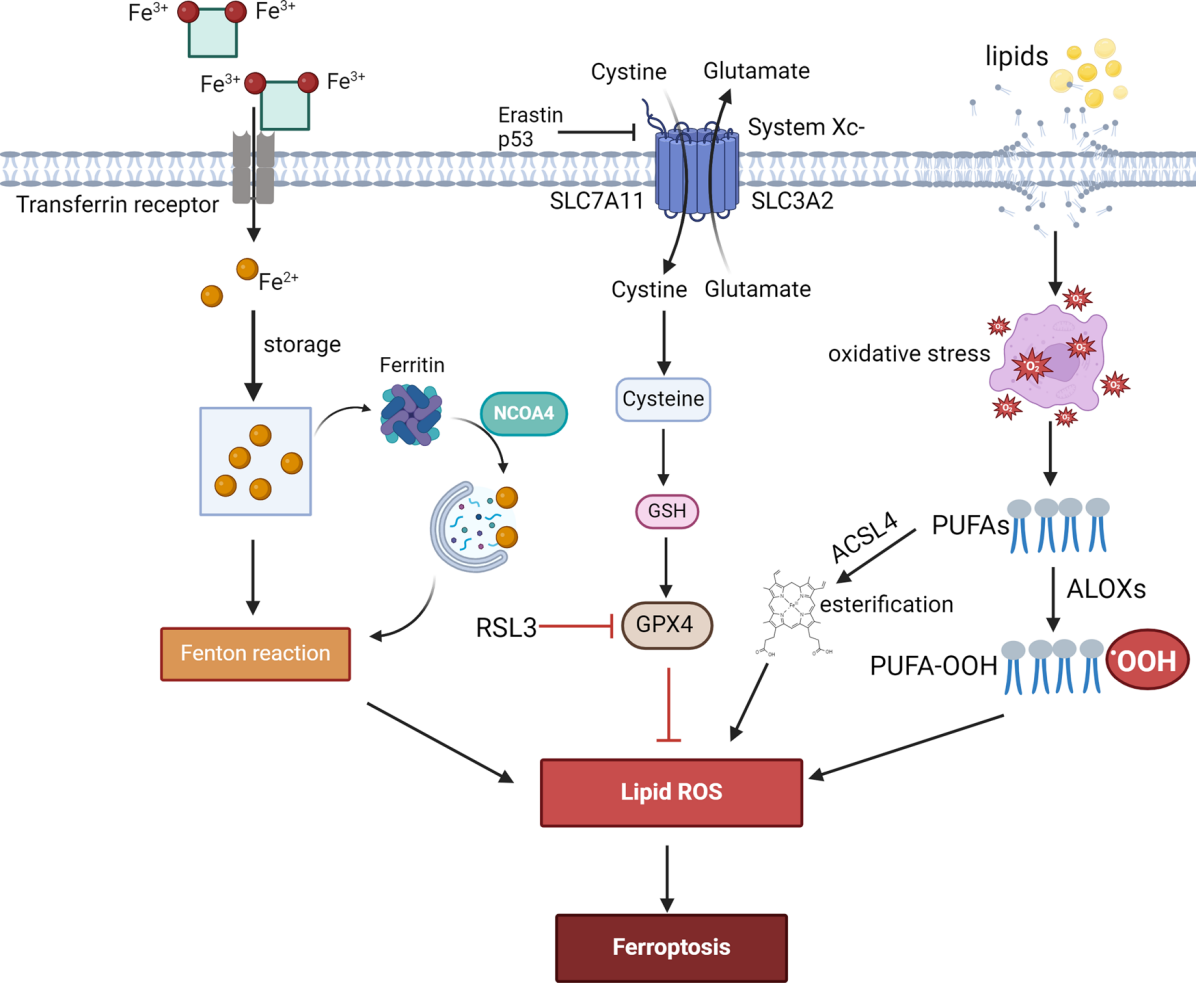
The process of ferroptosis involves three primary mechanisms. Firstly, lipid-mediated oxidative stress and subsequent membrane damage, as well as peroxidation of polyunsaturated (PUFAs) in phospholipids by lipoxygenases (ALOXs) activity, are crucial for ferroptosis. Secondly, System Xc− consists of solute carrier family 7 member 11 (SLC7A11) and solute carrier family 3 member 2 (SLC3A2), which regulate the entry and exit of cystine and glutamate into cells. The synthesis of GSH depends on cysteine availability, and cellular resistance to lipid oxidation relies on intracellular cysteine levels. GSH then reduces ROS and active nitrogen under the action of GPXs. Inhibiting the Xc− system affects GSH synthesis by inhibiting cystine absorption, leading to decreased GPX activity, reduced cellular antioxidant capacity, accumulation of lipid ROS, and ultimately oxidative damage and ferroptosis. Thirdly, extracellular iron ions bind to cell membrane protein transferrin (TF) in a trivalent form before binding to transferrin receptor (TFR) for endocytosis into lysosomes within the cell. The Fe^3+^ in the endosomes can be reduced to Fe^2+^, which can then bind with iron proteins, chelate with GSH free sulfhydryl groups, or be exported through the iron-transporting protein FPN. Excess Fe^2+^ accumulates within the cell, leading to the formation of unstable iron clusters. When cytoplasmic iron levels decrease, ferritin can release iron through ferritin autophagy facilitated by nuclear receptor coactivator 4 (NCOA4). Additionally, free Fe^2+^ in the unstable iron pool actively participates in the Fenton reaction, resulting in the generation of ROS, specifically hydroxyl radicals that contribute to membrane lipid peroxidation and ferroptosis.

In 2012, Dixon et al. coined the term “ferroptosis” to describe the cell death of cancer cells with RAS mutations induced by erastin [17]. The key feature of ferroptosis is the accumulation of lipid hydroperoxide, resulting from a decrease in glutathione peroxidases (GPXs). This process involves the interaction of free iron ions with lipid peroxides through the Fenton reaction, leading to the production of lipid free radicals and ultimately cell death. The main mechanisms underlying ferroptosis include lipid peroxidation, GSH synthesis and consumption, and abnormal iron metabolism (Fig. 1).

### Lipid peroxidation

Lipids play a crucial role in the regulation of cell death, with lipid-mediated oxidative stress and subsequent membrane damage being key factors leading to ferroptosis. The initial formation of lipid hydroperoxides (LOOHs) and the subsequent generation of reactive aldehydes (malondialdehyde, MDA, and 4-hydroxynonenal, 4HNE) are increased during ferroptosis due to lipid peroxidation. While various cell membrane lipids may undergo oxidation, the peroxidation of polyunsaturated fatty acids (PUFAs) in phospholipids by lipoxygenases (ALOXs) appears to be particularly important for ferroptosis [23]. Kim MW found that upregulation of elongation of very long-chain fatty acid protein 5 and fatty acid desaturase 1 sensitizes cells to ferroptosis [24]. Dierge et al. found that n-3 and n-6 PUFAs have been found to selectively induce ferroptosis in cancer cells under ambient acidosis [25]. In the ischemia phase of I/R, induction of ALOX15 triggers oxidization of PUFA-phospholipids, especially PUFA-phosphatidyl ethanolamines (PEs) leading to cardiomyocyte ferroptosis [26, 27]. Accumulation of intracellular lipid peroxides without timely elimination can cause oxidative damage to DNA, proteins, and the cell membrane ultimately leading to ferroptosis. Furthermore, modulation of the biosynthesis or catabolism of PUFAs will ultimately impact the cell’s susceptibility to ferroptosis. Acyl-CoA synthetase long-chain family member 4 (ACSL4), an enzyme involved in the activation of PUFAs and facilitation of esterification into arachidonic acid (AA) and adrenoyl into PE, plays a crucial role in promoting ferroptosis [28, 29]. AS-252424 (AS), a compound that acts as a potent inhibitor of ferroptosis, directly binds to glutamine 464 of ACSL4, inhibiting its enzymatic activity and suppressing lipid peroxidation [28]. The upregulation of ACSL4 expression by Sp1 suggests potential for extending ferroptosis inhibition to intestinal I/R treatment [30]. Inhibition of ACSL4 prevented radiation-induced ferroptosis in intestinal epithelial cells [31]. The specific role of ACSL4 in human diseases remains unclear, highlighting the need for better understanding its function and significance in relation to ferroptosis.

### GSH synthesis and consumption

The synthesis of GSH relies on cysteine as the starting material, and cellular resistance to lipid oxidation depends on intracellular cysteine levels, primarily produced by the system Xc− and transsulfuration pathways. System Xc− is an amino acid antiporter widely distributed in phospholipid bilayers and is part of an important antioxidant system in cells, consisting of two subunits, solute carrier family 7 member 11 (SLC7A11) and solute carrier family 3 member 2 (SLC3A2). Cystine and glutamate enter and exit the cell through the Xc− system in a 1:1 ratio [17].

GSH reduces ROS and active nitrogen under the action of GPXs. Inhibiting the activity of system Xc− affects GSH synthesis by inhibiting cystine absorption, leading to decreased GPX activity, reduced cellular antioxidant capacity, accumulation of lipid ROS, and ultimately oxidative damage and ferroptosis. Additionally, P53 can inhibit cystine uptake through downregulation of SLC7A11 expression within the Xc− system, affecting GPX4 activity which leads to reduced cellular antioxidant capacity, accumulation of lipid ROS, and ferroptosis [32, 33]. GPX4, a member of the GPX family, plays a crucial role in regulating cell ferroptosis by primarily inhibiting the formation of lipid peroxides. Inhibition of GPX4 activity can result in the accumulation of lipid peroxides, which is a characteristic feature of ferroptosis. Cells with downregulated GPX4 expression are more susceptible to ferroptosis, whereas high levels of GPX4 can effectively suppress this process [34, 35].

### Abnormal iron metabolism

Iron is a crucial trace element for the human body, and its deficiency can result in anemia and abnormalities in iron-dependent enzymes. Conversely, the accumulation of iron can lead to tissue damage and increase the risk of developing various diseases such as tumors [36]. Reversely, the cell membrane protein transferrin (TF) typically binds extracellular iron ions in a trivalent form. After that, they are taken up by the transferrin receptor (TFR) through endocytosis and enter the cell lysosome. Inside the cell, metal reductase six-transmembrane epithelial antigen of prostate 3 reduces trivalent iron ions to Fe^2+^, which are then released from the lysosome into the cytoplasm via solute carrier family 11 member 2 for participation in various subsequent physiological and biochemical processes [37, 38].

There are three ways for Fe^2+^ to enter cells: it can bind with iron proteins, chelate with GSH free sulfhydryl groups, or be exported through the iron-transporting protein solute carrier family 40 member 1 (FPN). Excess Fe^2+^ accumulates within the cell, leading to the formation of unstable iron clusters. When cytoplasmic iron levels decrease, ferritin is able to release iron through ferritin autophagy, a process that is facilitated by nuclear receptor coactivator 4 (NCOA4) [39]. Reactive Fe^2+^ in the labile iron pool is involved in the Fenton reaction, leading to the production of ROS, particularly hydroxyl radicals that contribute to oxidative damage of membrane lipids and ferroptosis [37]. The classical ferroptosis activators erastin or RSL3 primarily inhibit the antioxidant system by increasing accumulation [17]. Iron can directly produce excess ROS in cells through Fenton reaction and increase cellular oxidative damage. Additionally, iron can increase ALOX or EGLN proline hydroxylase (also known as PHD) activity, affecting cellular lipid peroxidation and oxygen homeostasis and thereby influencing cellular ferroptotic processes [40].

### Overview of *Helicobacter pylori* virulence factors related with ferroptosis

CagA and VacA are the most extensively studied virulence factors of *H. pylori*, and they have been implicated in the development of GC. DNA microarray technology has identified differentially regulated genes when *H. pylori* is cultured under iron starvation conditions, revealing the inclusion of known virulence genes such as CagA, VacA, and NapA [41]. Studies have also found a close relationship between NapA, SodB, and ferritin, which plays a role in storing Fe^3+^ and preventing its availability to promote ferroptosis [42]. Additionally, γ-glutamyl transpeptidase (gGT), lipopolysaccharide (LPS) and outer inflammatory protein A (OipA) could affect GPX4 and Xc− expression then regulated ferroptosis. Therefore, this review primarily focuses on the functions of these virulence factors of *H. pylori* in relation to ferroptosis (Table 1).

**Table 1:** Virulence factors of *H. pylori*-associated with ferroptosis.

Virulence factors	Effect	Reference
CagA	promotes the synthesis of PUFA-ePLs	[49]
	NOX4/NRF2/GPX4 pathway mediates CagA	[20]
	increases iron uptake through transferrin endocytosis and decreases cytoplasmic labile iron pool while increasing lysosomal iron	[50]
	iron deficiency could enhance the virulence of *H. pylori*	[50]
VacA	elevates ROS levels	[55–57]
	impairs GSH metabolism	[58, 59]
Nap	Nap binds Fe^2+^ ions, which are further oxidized to Fe^3+^	[61, 62, 67]
SodB	FecA1 is an essential gene for SodB activation	[69]
	Fur is a direct regulator of SodB	[71]
gGT	catabolize reduced GSH into glutamate and cysteinylglycine (Cys-Gly)	[72]
LPS	increased GPX4 expression and elevated ROS production	[73]
OipA	decreased Xc− expression	[74]

### Cytotoxin-associated gene A (CagA)

The primary virulence factors of *H. pylori*, CagA and the type 4 secretion system (T4SS), are encoded by the cytotoxin-associated gene pathogenicity island (cagPAI) and play a crucial role in carcinogenesis [43]. Injection of CagA into cells through T4SS fimbriae induces cell changes, impairs cell motility, proliferation, apoptosis, and alters cytoskeleton arrangement. *H. pylori* infection induces GC cells to express L-plastin via CagA-activated mitogen-activated protein kinase 1 (ERK) signaling pathway to mediate SP1 binding to L-plastin promoter, which promotes proliferation and migration [44]. Additionally, CagA may contribute to the development of GC by subverting a Wnt-dependent planar cell polarity-dependent mechanism that restrains pyloric gland stem cell proliferation and promotes enteroendocrine differentiation [45]. As an important virulence factor of *H. pylori*, CagA can regulate the progression of GC in various ways. Researchers have also discovered that CagA can influence lipid metabolism and iron content, thereby regulating the development of GC.

Noto [46] discovered that iron deficiency can enhance the formation of *H. pylori* cag type IV secretion system and facilitate CagA entry into host cells, leading to gastric carcinogenesis. Iron deficiency may increase the virulence of *H. pylori*, serving as a measurable biomarker for identifying populations at high risk for GC [47, 48]. CagA modulates host cell iron homeostasis and fundamental metabolic functions of the bacterial cell, promoting the synthesis of polyunsaturated ether phospholipids (PUFA-ePLs) through increased expression of alkylglycerone phosphate synthase and 1-acylglycerol-3-phosphate O-acyltransferase 3, resulting in susceptibility to ferroptosis in GC cells [49]. Myricetin regulates the inhibition of ferroptosis induced by CagA through the NADPH oxidase 4 (NOX4)/nuclear respiratory factor 2 (NRF2)/GPX4 pathway [20]. The CagA protein increases iron uptake through TF endocytosis and decreases cytoplasmic labile iron pool while increasing lysosomal iron through enhanced expression of H-ferritin in *H. pylori*-infected AGS cells. The observed increase in lysosomal labile iron observed in *H. pylori*-infected AGS cells results in improved growth of *H. pylori* [50]. Overall, CagA mediates cellular ferroptosis through its influence on iron and ROS content, lipid oxidation, and GPX4 activity.

### Vacuolating cytotoxin A (VacA)

VacA, a renowned virulence factor of *H. pylori*, elicits a array of detrimental effects on host cells, including vacuolation, disruption of lysosomal function, and modulation of the immune response [51, 52]. Additionally, this protein modulates mitochondrial function and orchestrates apoptosis, autophagy and necrosis [53, 54]. VacA elevates ROS levels, and the antioxidant N-acetyl-L-cysteine abrogates this effect, consequently suppressing autophagy [55]. *H. pylori* VacA reduces GSH levels leading to ROS accumulation and activation of protein kinase B (AKT) induced autophagy and CagA degradation. Furthermore, CagA specifically accumulates in gastric cells expressing CD44 exhibiting resistance to ROS resulting in increased intracellular GSH levels that can suppress the autophagic pathway [56]. VacA-deficient *H. pylori* upregulates integrin linked kinase (ILK) expression modulating endothelial nitric oxide synthase (eNOS) expression promoting ROS production enabling *H. pylori* to evade the host immune response, contributing to the persistent infection in the stomach [57]. The outer membrane vesicles (OMVs) containing VacA-treated AGS cells induced micronuclei formation accompanied by alterations in iron metabolism and GSH depletion [58]. Also, VacA impairs GSH metabolism in gastric epithelial cells weakening their resistance against oxidative stress or cellular redox regulation by GSH [59]. These studies indicate that VacA may contribute to the ferroptosis in GC progression by increasing ROS levels and disrupting the metabolism of GSH through various mechanisms, but there is no reports on the morphology and related proteins of ferroptosis cells with VacA, which is worthy of further investigation.

### Neutrophil-activating protein (Nap)

Nap, a virulence factor of *H. pylori*, induces adhesion of neutrophils to gastric epithelial cells and promotes ROS and myeloperoxidase production primarily during the quiescent phase of infection. It activates neutrophils and mast cells, as well as promotes monocyte migration [60]. Nap exhibits pro-inflammatory activity and plays a significant role in the progression of inflammation and tissue damage during *H. pylori* infection. Structurally similar to ferritin, Nap possesses iron oxide enzyme activity but does not directly bind with iron oxide enzyme. With iron oxidase centers, Nap binds Fe^2+^ ions which are further oxidized to Fe^3+^, generating hydroxyl radicals that protect *H. pylori* DNA from damage [61, 62]. In addition to iron ions, Nap also has the capacity to sequester other ions such as zinc or cadmium [63].

*H. pylori*-Nap directly interacts with Toll-like receptor 2 (TLR2) and activates it to induce IL-8 secretion in neutrophils and all-trans retinoic acid-induced differentiated HL-60 cells [64]. ROS and low concentrations of hydrogen peroxide (H_2_O_2_) can promote the formation of *H. pylori* biofilm. While NapA further promotes H_2_O_2_-induced biofilm formation and confers multidrug resistance. Additionally, vitamin C exhibits anti-*H. pylori* biofilm activity and downregulates the expression of napA [65]. *H. pylori* infection and Nap may contribute to the pathogenesis of anti-aquaporin-4 antibody-related neural damage by activating neutrophils [66]. *H. pylori* strains with Thr70-type NapA have been found to enhance Fe ion uptake ability in cases of iron-deficiency anemia [67]. While NapA possesses pro-inflammatory and iron storage capabilities, its potential role in ferroptosis in GC and related human malignancies has not been extensively studied, thus warranting further consideration and exploration.

### Superoxide dismutase B (SodB)

*H. pylori* encodes a single iron-cofactored SodB, which is regulated by the ferric uptake regulator (Fur), an Fe^2+^-dependent transcriptional repressor. Fe^2+^ is required for the activation of SodB [68, 69]. The Fe^3+^-dicitrate transporter homolog, fecA1, is essential for SodB activation but not for the biogenic activity of *H. pylori*. Deletion of fecA1 results in reduced resistance to H_2_O_2_, decreased gastric mucosal-colonization ability in Mongolian gerbils, and lowered resistance to metronidazole (Mtz) in *H. pylori* with inactive SodB. FecA1 plays a crucial role in host-colonization ability and Mtz resistance through Fe^2+^ supply to SodB, suggesting that it may be a potential target for novel bactericidal drug development [69]. Nordihydroguaiaretic acid (NDGA) decreases intracellular Fe^2+^ levels in *H. pylori* and inhibits SodB activity. Additionally, NDGA enhances H_2_O_2_ sensitivity and increases Mtz sensitivity in *H. pylori* [70]. Fur directly regulates the iron-cofactored superoxide dismutase SodB in *H. pylori*, which is essential for defense against toxic superoxide radicals [71]. These studies indicate that FecA1 can provide Fe^2+^ to SodB, play an important role in the colonization of the host and Mtz resistance of *H. pylori*, and provide a new therapy for the eradication of *H. pylori*.

### γ-glutamyl transpeptidase (gGT)

γ-glutamyl transpeptidase (gGT) is essential for *H. pylori* colonization and serves as a well-established virulence factor with immunomodulatory properties. *H. pylori* utilizes gGT to catabolize reduced GSH into glutamate and cysteinylglycine (Cys-Gly) from gastric cells, while also internalizing Cys-Gly in a gGT-dependent manner [72]. It is proposed that the influence of gGT on Cys-Gly production in *H. pylori* infection may be linked to a decrease in intracellular GSH levels in infected cells, suggesting a potential impact of gGT on GPX4 expression and ferroptosis in *H. pylori* infected cells.

### Lipopolysaccharide (LPS)

*H. pylori* LPS treatment of AGS cells led to an increase in toll-like receptor 4 (TLR4), GPX2 and GPX4 expression, as well as elevated ROS production, subsequently altering the level of IL-8 expression [73]. This suggests that *H. pylori* LPS impacts GPX4 expression and ROS production, but there is currently no relevant study on its role in cells ferroptosis, indicating a need for further exploration of the function of *H. pylori* LPS in ferroptosis.

### Outer inflammatory protein A (OipA)

The recombinant OipA protein of *H. pylori* decreased Xc− expression and increased miR-30b expression, OipA reduced the protective effects of the Xc-/glutamate pathway on gastric mucosa by regulating miRNA-30b [74]. Xc− system is crucial in regulating ferroptosis and SLC7A11 facilitates the exchange of extracellular cystine and intracellular glutamate across the plasma membrane, as well as the reduction of cystine to cysteine within cells, which is essential for GSH production [75]. *H. pylori* OipA has been shown to impact the Xc− system, but its direct influence on the ferroptosis process of gastric mucosa cells remains unexplored, presenting a significant research gap that warrants further investigation.

## Role of pathogen-*H. pylori* in ferroptosis of gastric cancer

**Fig. 2:**
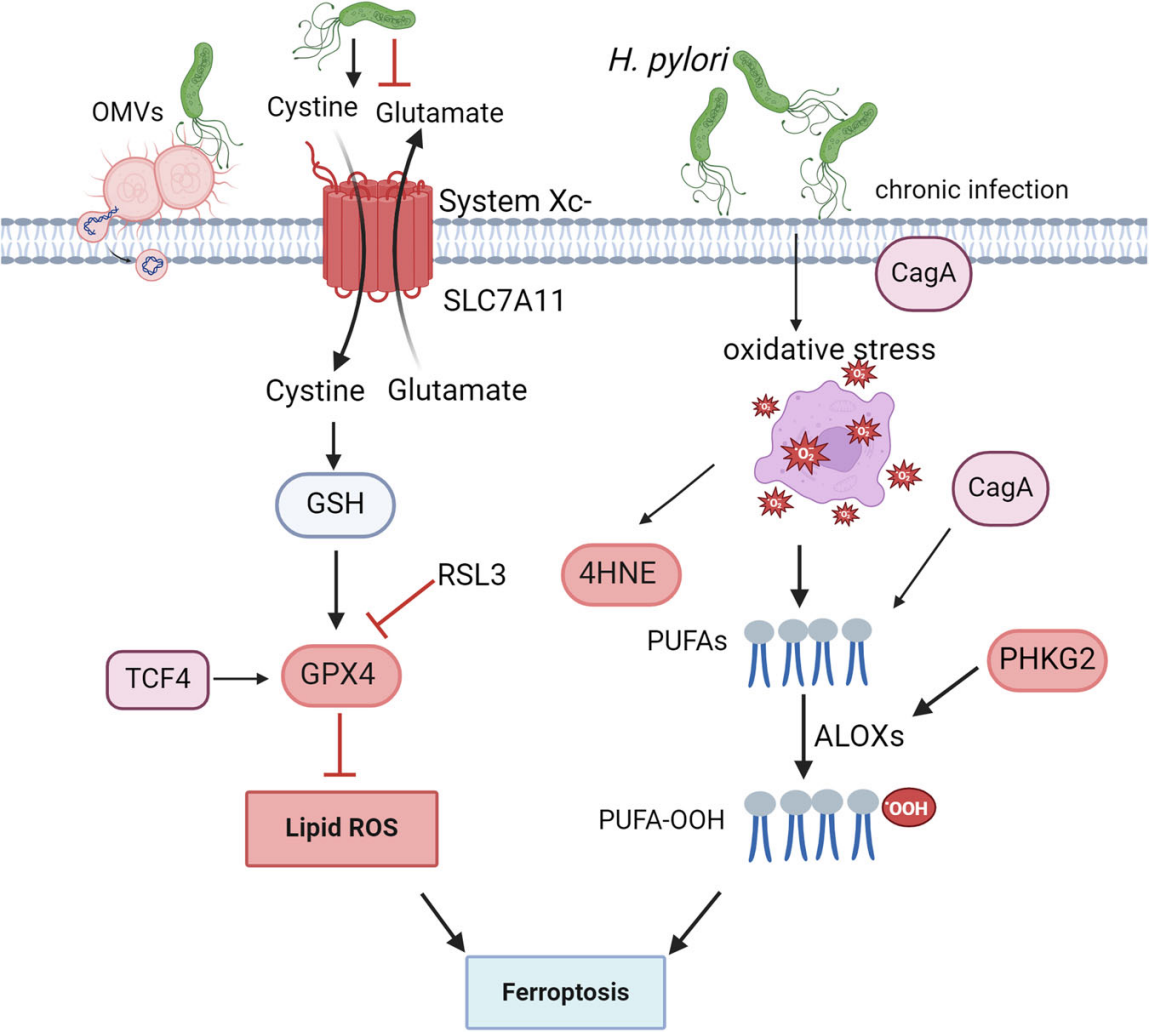
The function of ferroptosis in *H. pylori* infection gastric cancer. *H. pylori* infection could be up-regulated or downregulated in gastric mucosal cells. *H. pylori* mediated the expression of TCF4 and then regulated GPX4 and ROS, influenced cell ferroptosis. Also, *H. pylori* and its infection-regulated PHKG2 expression mediated ALOXs influenced ferroptois. Furthermore, the OMVs and CagA all play an important role in the regulation of ferroptosis mediated by *H. pylori*.

Studies have found that a variety of pathogenic bacteria can regulate ferroptosis processes to affect disease progression. Qiang et al. [76] found tuberculosis (TB) treatment via blocking Mycobacterium tuberculosis (Mtb) protein tyrosine phosphatase A -host protein arginine methyltransferase 6 interface to target GPX4-dependent ferroptosis, eventually inducing ferroptosis to promote Mtb pathogenicity and dissemination. Mtb infection of macrophages resulted in decreased levels of GSH and GPX4, increased free iron, mitochondrial superoxide, and lipid peroxidation. Also, infected animals treated with ferrostatin-1 (fer-1) demonstrated a reduction of bacterial number [77]. These studies suggested that pathogenic bacteria can regulate ferroptosis mediate disease progression. By utilizing various virulence factors, such as CagA and its Cag PAI, and VacA, *H. pylori* targets different cellular proteins to modulate the host inflammatory response and trigger pathological reactions to gastric mucosa, leading to chronic gastritis and peptic ulcer. Although *H. pylori* virulence factors have a causal relationship with the development of GC, not all people with *H. pylori* or gastric precancerous lesions will progress to GC. Therefore, the study of host factors affecting gastric environment and the occurrence of GC has attracted extensive attention. We summarizes the effects of *H. pylori* on the progression of GC through ferroptosis, and explores the main mechanism of its effects (Fig. 2).

### *H. pylori* affected GSH synthesis and consumption

*H. pylori* 7.13, PMSS1, and ATCC 43504 infected with AGS cells increased SLC7A11 expression [78, 79]. Treatment with selenocystine and ebselen (500 μg/kg/day) resulted in significant reduction in ROS production and inhibition of lipid peroxidation, induced GPX4 expression in gastric tissue that induced ulcers in rodent model infected with *H. pylori* [80]. *H. pylori* induced cell apoptosis with a decrease in glutamate release and Xc− activity in cultured GES-1 cells, and these effects of *H. pylori* were attenuated by Xc- (SLC7A11) overexpression. In mice, *H. pylori* infection induced gastric mucosal injury with downregulation of Xc- expression [74]. *H. pylori* 26695 and 11637 upregulates GPX4 expression and activity via Transcription Factor 4 (TCF4), leading to the regulation of lipid peroxidation levels. Eradication of *H.pylori* can relatively enhance chemo-sensitivity in *H. pylori*-positive GC patients by triggering ferroptosis [81]. However, researchers found that *H. pylori* infected gastric adenocarcinoma cells AGS increased SLC7A11 expression, while decreased SLC7A11 expression in normal gastric mucosa GES-1 cells, whether this is related to differences between *H. pylori* strains and host cells, or to other factors is controversial and needs further investigation.

### *H. pylori*-mediated lipid peroxidation affected ferroptosis

Ferroptosis inhibitors have demonstrated benefits in certain diseases due to their anti-inflammatory activity [82]. *H. pylori* infection increased the sensitivity of GC cells to RAS-selective lethal 3 (RSL3)-induced ferroptosis, and phosphorylase kinase G2 (PHKG2) expression was significantly associated with *H. pylori* infection. In addition, *H.pylori* induces PHKG2 to regulate the lipoxygenase ALOX5, which is the mechanism of cell sensitivity to ferroptosis [83]. Furthermore, CagA promotes the synthesis of PUFA-ePLs, which is mediated by increased expression of alkylglycerone phosphate synthase and 1-acylglycerol-3-phosphate O-acyltransferase 3, leading to susceptibility to ferroptosis [49]. 4‐HNE and MDA were lipid peroxidation markers [84, 85], 4-HNE targets GPX4 for ubiquitin-related degradation induced ferroptosis [86], found that *H. pylori*-induced oxidative stress, and increased the levels of intracellular ROS and 4-HNE [87, 88]. Above all, *H. pylori* influenced lipid peroxidation pathway affects ferroptosis.

### Others elements

The OMVs of *H. pylori* altered the gene expression profile of gastric epithelial cells, and the most abundant pathways of the OMVs from *H. pylori* included the pathways related to autophagy and ferroptosis, providing a new direction for the exploration of *H. pylori* mediated diseases [89]. After comprehensive analysis of YWHAE gene in GC, it was found that YWHAE was closely linked with *H. pylori* infection and ferroptosis in GC [90]. In addition, four ferroptosis-related genes: NOX4, MTCH1, GABARAPL2, and SLC2A3, were identified and shown to accurately predict GC and *H. pylori*-associated GC, the ferroptosis inducer FIN56 inhibited the expression in MKN-45 and HGC-27 cells [91]. In the above studies, *H. pylori* and other components such as OMVs, acted on gastric mucosal cells or tissues, bioinformatics analysis found that it could regulate the ferroptosis pathway, but its specific mechanism of action still needs to be further explored.

## Drugs therapy targeting ferroptosis in *H. pylori-*infected gastric cancer

Recent studies have shown that ferroptosis plays a significant role in the development of GC and *H. pylori* infection. Understanding the functions of ferroptosis inducers, inhibitors, and drugs in *H. pylori*-infected GC is crucial for developing effective therapeutic strategies against GC, especially in the context of *H. pylori* infection.

### Ferroptosis inducers in *H. pylori-*infected gastric cancer

Reagents that induce ferroptosis, such as erastin, sulfasalazine, and glutamate, block the system Xc- and hinder the uptake of cysteine by cells. RSL3, DPI10, DPI13, ML162 and ML210 promote ferroptosis by inhibiting GPX4, while FIN56 leads to degradation of GPX4 [92, 93]. *H. pylori* infection raises the vulnerability of cells to ferroptosis, and PHKG2 boosts RSL3-triggered ferroptosis in *H. pylori*-positive GC cells by stimulating ALOX5 expression [83]. The CagA deletion mutant strain exhibits reduced sensitivity to ferroptosis inducers. Treatment with the ferroptosis inhibitors deferoxamine (DFO) and liproxstatin-1 can rescue the sensitivity of CagA-induced cells to RSL3 and erastin, suggesting a potential therapeutic strategy for inducing ferroptosis in GC patients infected with CagA+ *H. pylori* strain [49]. *H. pylori*-infected GC cells enhance glucocerebrosidase (GBA1) expression; 10 μM concentration of erastin regulates ferroptosis sensitivity in GC cells by *H. pylori* via GBA1 [94]. These studies suggest that the ferroptosis inducer erastin and RSL3 may play a role in the treatment of *H. pylori* infection.

Knockdown of B cell receptor-associated protein 31 (BAP31) increases the susceptibility of GC cells to erastin, suggesting that BAP31 may serve as a prognostic factor for GC and a potential therapeutic strategy [95]. HGC-27 cells exhibit heightened sensitivity to erastin upon overexpression of cytoplasmic polyadenylation element binding protein 1 (CPEB1), which in turn reduces twist1 expression and activates the ATF4/ChaC glutathione-specific gamma-glutamylcyclotransferase 1 (CHAC1) pathway [96]. Erastin-induced ferroptosis via the Xc-mediated ROS/P38-MAPK pathway feedback loop presents novel strategies for comprehensive treatment of GC [97]. Low dose of erastin inhibit malignant behavior and induce apoptosis by causing mitochondrial dysfunction in GC cells [98]. Inhibition of NIMA-related kinase 2 (NEK2) enhances the sensitivity of GC cells to RSL3 and erastin-induced ferroptosis [21]. ATF3 overexpression, combined with treatment with erastin or RSL3, enhances ferroptosis and cisplatin resistance in GC cells [99]. Additionally, NOP2/Sun RNA methyltransferase 5 (NSUN5) - ferritin heavy chain (FTH1) axis regulates erastin-induced ferroptosis in SGC-7901 cells; NSUN5 and FTH1 promote GC cell growth partly through suppression of ferroptosis [100]. The mRNA and protein levels of the hub genes decrease in a dose-dependent manner in FIN56-treated GC cells, indicating that FIN56 regulates hub gene expression during ferroptosis and suggesting its potential as a drug for inhibiting invasion by GC cells [91]. The aforementioned studies indicate that a ferroptosis inducer may serve as a promising pharmaceutical agent for the treatment of GC.

### Ferroptosis inhibitors in *H. pylori-*infected gastric cancer

Ferroptosis inhibitors, such as fer-1, liproxstatin-1, zileuton, α-tocopherol, FSP1, BH4 and CoQ10, interrupt the lipid peroxidation cascade. Additionally, DFO, deferiprone and N-acetylcysteine (NAC) have also been classified as ferroptosis inhibitors due to their targeting of other cellular pathways [92]. However, there is currently a lack of relevant research on ferroptosis inhibitors in *H. pylori-*infected GC despite its significant role in the progression of GC.

Apatinib induced ferroptosis which was blocked by co-incubation with fer-1 and liproxstatin-1 in GC cells [101]. Furthermore, deletion of lysyl oxidase-like 3 (LOXL3) resulted in the activation of ferroptosis in GC cells; however this suppressive effect was compensated by treatment with fer-1 [102]. Dexmedetomidine (DEX) induced ferroptosis but this effect was abolished by treatment with fer-1 in GC cells [103]. Functional targeting of cancer-associated fibroblasts (CAFs) using a combination of DFO and follistatin-like protein 1 (FSTL1) -neutralizing antibody significantly alleviated CAF-induced natural killer cell (NK cell) ferroptosis and boosted the cytotoxicity of NK cells against GC [104]. The novobiocin derivative XN4 increased ferroptosis, and the effect was reverse by treatment with DFO in GC cells [105]. Compound a2 markedly elevated the level of ROS; however ROS accumulation triggered by a2 was almost completely reversed by the ROS scavenger NAC [106]. While there are no reports on the impact of ferroptosis inhibitors on *H. pylori* infection progression, fer-1, liproxstatin-1, DFO, and NAC can influence GC progression through various mechanisms and plays a crucial role in its development.

### Other drugs in *H. pylori-*infected gastric cancer

Reaching the goal of developing new anticancer drugs for clinical use by targeting ferroptosis is a time-consuming process. However, recent research has indicated that numerous clinically approved drugs demonstrate strong anti-tumor effects by either promoting or suppressing non-apoptotic regulated cell death mechanisms. Studies found that sorafenib, sulfasalazine, and slutamate could inhibit Xc-system affect ferroptosis in human cancer [17, 107, 108]. There is no relevant study on sulphasalazine with *H. pylori*-infected GC, but the treatment further decreased the prevalence of *H. pylori* of patients with inflammatory bowel disease and Crohn’s disease [109]. In addition, octreotide and cisplatin plays a role for GPX4 inactivation for human cancer [110, 111], ultra-short octreotide containing quadruple therapy is a safe and effective regime in eradicating *H. pylori* and healing peptic ulcers [112]. There is no significant benefits on octreotide in patients with acutely bleeding benign peptic ulcer or/and visible vessel regarding their outcome [113]. No clinical trials have investigated the impact of sorafenib, glutamate and cisplatin on *H. pylori*-related conditions, and ferroptosis represents a promising therapeutic endpoint for cancers.

## Conclusion

In recent years, the significance of ferroptosis in human progress has become a topic of increasing interest. This review not only summarized the main mechanisms of ferroptosis, but also concluded the role of *H. pylori* and its virulence factors on ferroptosis.

The process of ferroptosis is regulated by lipid peroxidation, GSH synthesis and consumption, and abnormal iron metabolism [40, 81], and *H. pylori* and its virulence factors can influence ferroptosis by regulating different ways. CagA mainly affected PUFA-ePLs, GPX4 expression and iron ion [43, 46, 49], VacA mainly regulate the GSH metabolism and ROS levels [52, 57, 58]. NapA mainly affect the storage and release of cellular Fe^2+^ [41, 65, 67]. By suppressing intracellular Fe^2+^ uptake by FecA1 and thereby reducing SodB activity associated with gastric mucosal-colonization [69, 70]. *H. pylori* gGT reduced GSH into glutamate and Cys-Gly from gastric cells [72], *H. pylori* LPS could increase the expression of GPX4 that was negatively regulates ferroptosis [73]. *H. pylori* OipA decreased Xc- expression [74]. *H. pylori* virulence factors can regulate different ways of ferroptosis and affect disease progression, and the bacteria itself can also affect lipid metabolism [83], GPX4 expression [80, 81], Xc- system [74, 78] and other regulatory processes of ferroptosis. However, the impact of *H. pylori* on Xc- expression is complex and warrants further investigation. Additionally, the potential influence of outer membrane proteins and adhesins, as important virulence factors of *H. pylori*, on the ferroptosis process in GC cells has not been explored. Furthermore, there is a research gap regarding the role of *H. pylori* in iron metabolism and its regulation of ferroptosis. Therefore, this review suggests that further study into the influence of *H. pylori* and its virulence factors on ferroptosis in GC could provide a new direction for treating *H. pylori* infection.

*H. pylori* infection and CagA have been found to suppress ferroptosis, while erastin and RSL3 are capable of inducing ferroptosis in the treatment of GC infected with *H. pylori*, ferroptosis inducers presents a novel strategy for comprehensive GC treatment. It is worth noting that ferroptosis inhibitors have been shown to exacerbate the progression of GC, further underscoring the significance of targeting ferroptosis in GC treatment. Furthermore, other drugs such as Sorafenib, Glutamate, sulfasalazine, octreotide, and cisplatin have demonstrated effects on the Xc- system and GPX4. The treatment of sulphasalazine decreased the prevalence of *H. pylori* of patients with inflammatory bowel disease and Crohn’s disease [109] and ultra-short octreotide containing quadruple therapy is a safe and effective regime in eradicating *H. pylori* and healing peptic ulcers[112]. Limited research has been conducted on specific iron-targeting drugs for treating *H. pylori* infection-related diseases in clinical trials.

Exploration into the specific mechanisms and pathways involved in inhibiting ferroptosis could offer valuable insights into developing innovative therapeutic strategies for *H. pylori* infection. Overall, this review suggest that inhibiting ferroptosis may contribute to the progression of gastric cancer, more research is necessary to fully comprehend its therapeutic potential in the context of *H. pylori* infection.

## Data Availability

No data was used for the research described in this article.
